# The increase in medial prefrontal glutamate/glutamine concentration during memory encoding is associated with better memory performance and stronger functional connectivity in the human medial prefrontal–thalamus–hippocampus network

**DOI:** 10.1002/hbm.24008

**Published:** 2018-02-27

**Authors:** Jan‐Willem Thielen, Donghyun Hong, Seyedmorteza Rohani Rankouhi, Jens Wiltfang, Guillén Fernández, David G. Norris, Indira Tendolkar

**Affiliations:** ^1^ Erwin L. Hahn Institute for Magnetic Resonance Imaging Essen Germany; ^2^ Donders Institute for Brain Cognition and Behavior, Radboud University and Radboud University Medical Center Nijmegen the Netherlands; ^3^ Department for Psychiatry and Psychotherapy, Faculty of Medicine University of Duisburg‐Essen Essen Germany; ^4^ Department of Psychiatry and Psychotherapy University Medical Center Göttingen Göttingen Germany; ^5^ Department of Cognitive Neuroscience Radboud University Medical Center Nijmegen the Netherlands; ^6^ MIRA Institute for Biomedical Technology and Technical Medicine, University of Twente Enschede the Netherlands; ^7^ Department of Psychiatry Radboud University Medical Center Nijmegen the Netherlands

**Keywords:** fMRI, functional connectivity, GABA, glutamate, hippocampus, medial prefrontal, memory, MR spectroscopy, network, thalamus

## Abstract

The classical model of the declarative memory system describes the hippocampus and its interactions with representational brain areas in posterior neocortex as being essential for the formation of long‐term episodic memories. However, new evidence suggests an extension of this classical model by assigning the medial prefrontal cortex (mPFC) a specific, yet not fully defined role in episodic memory. In this study, we utilized 1H magnetic resonance spectroscopy (MRS) and psychophysiological interaction (PPI) analysis to lend further support for the idea of a mnemonic role of the mPFC in humans. By using MRS, we measured mPFC γ‐aminobutyric acid (GABA) and glutamate/glutamine (GLx) concentrations before and after volunteers memorized face–name association. We demonstrate that mPFC GLx but not GABA levels increased during the memory task, which appeared to be related to memory performance. Regarding functional connectivity, we used the subsequent memory paradigm and found that the GLx increase was associated with stronger mPFC connectivity to thalamus and hippocampus for associations subsequently recognized with high confidence as opposed to subsequently recognized with low confidence/forgotten. Taken together, we provide new evidence for an mPFC involvement in episodic memory by showing a memory‐related increase in mPFC excitatory neurotransmitter levels that was associated with better memory and stronger memory‐related functional connectivity in a medial prefrontal–thalamus–hippocampus network.

## SIGNIFICANCE STATEMENT

In this study, we combined 1H magnetic resonance spectroscopy (MRS) and functional magnet resonance imaging (fMRI) to lend further support for a role of the medial prefrontal cortex (mPFC) in episodic memory. The combination of MRS and fMRI can provide deeper insight in the neuronal correlates of episodic memory as MRS offers additional information in the dynamics of biochemistry in an activated brain. By combining these measurements, we were able to provide new evidence for an mPFC involvement in episodic memory by showing a memory‐related increase in excitatory neurotransmitter levels that was associated with better memory and stronger memory‐related functional connectivity in a memory network.

## 1 INTRODUCTION

The literature on the medial prefrontal cortex (mPFC) is dominated by studies regarding its role in decision making (Euston, Gruber, & McNaughton, 2012), but recent evidence suggests that the mPFC may also play a role in memory (Szczepanski and Knight, 2014; Preston and Eichenbaum, 2013; van Kesteren, Rijpkema, Ruiter, & Fernández, 2013; Euston et al., 2012; van Kesteren, Ruiter, Fernandez, & Henson, 2012; Takashima et al., 2006; Bontempi, Laurent‐Demir, Destrade, & Jaffard, 1999). The mPFC appears to serve as a critical hub obtaining, integrating and applying knowledge for long‐term use (Fernández, 2017). This role is supported by anatomical connections revealed in nonhuman primates and rodents showing that the mPFC receives unidirectional afferents from the hippocampus (Aggleton, Dumont, & Warburton, 2011; Insausti and Munoz, 2001; Barbas and Blatt, 1995) and in turn, has reciprocal connections to several thalamic nuclei including anterior, medial dorsal, and midline nuclei, which are indirectly or directly reciprocally connected to the hippocampus (Aggleton et al., 2011). Thus, the unidirectional connection from hippocampus to mPFC may be reciprocated via routes through the thalamus. Indeed, a recent rodent study showed that the mPFC controls the excitability of hippocampal neurons via midline thalamic nuclei during fear memory encoding (Xu and Südhof, 2013), and in humans, it has been shown that midline thalamic nuclei mediate between mPFC and hippocampus during high‐confidence memory retrieval (Thielen, Takashima, Rutters, Tendolkar, & Fernández, 2015). Previous imaging studies have already shown that successfully and confidentially remembered associations lead to increased activation in hippocampal and medial prefrontal cortices during encoding (Sperling et al., 2003; Chua, Rand‐Giovannetti, Schacter, Albert, & Sperling, 2004; Chua, Schacter, Rand‐Giovannetti, & Sperling, 2007).

Functional MRI, however, offers only indirect measures of neural activity and does not disclose the underlying biological mechanism. Neural activation that is related to GABAergic and glutamatergic transmission induces different cellular processes that result in long‐lasting synaptic alterations as, for instance, long‐term potentiation (LTP). Animal and *in vitro* data have shown that manipulating GABAergic and glutamatergic transmission via receptor specific ligands has specific effects on LTP (Lüscher and Malenka 2012; Maffei 2011; Kullmann & Lamsa, 2011). In this regard, it has been shown that blocking glutamatergic transmission causes LTP reduction and impaired learning (Davis, Butcher, & Morris, 1992; Stäubli, Rogers, & Lynch, 1994) whereas blocking GABAergic transmission facilitates LTP and learning (Stäubli, Scafidi, & Chun, 1999; Kalueff and Nutt 1997). Moreover, in animals, it has been shown that forebrain concentrations of glutamate and glutamine were increased after passive avoidance learning (Hertz, O'Dowd, Ng, & Gibbs, 2003) indicating glutamate/glutamine synthesis during learning. The detection of such a specific modulation of neurotransmitter concentrations in the human mPFC would lend further support for the proposal that it serves a mnemonic role because it may link it to neural plasticity and not just activity. Recent progress in MR spectroscopy appears to enable measuring learning‐related changes in neurotransmitter concentrations. By utilizing 1^H^MR‐spectroscopy, it has been shown that learning novel motor skills decreases GABA concentrations in the motor cortex (Floyer‐Lea, Wylezinska, Kincses, & Matthews, 2006; Sampaio‐Baptista et al., 2015) suggesting a potential role of GABA reactivity in associative learning. Also with respect to the mPFC some initial evidence is reported. Michels et al. (2012) showed a decrease in GABA concentration after a working memory task, whereas Huang et al. (2015) revealed an increase in glutamate/glutamine (GLx) when subjects engage in a mental imagery task. To date, however, evidence is lacking whether changes in medial prefrontal neurotransmitter levels are more clearly associated with memory and whether such effects are related to the mPFC–thalamus–hippocampus network.

In light of these aforementioned findings, the present study aimed at probing associative memory networks in the human mPFC–thalamus–hippocampus network by utilizing functional connectivity analysis and its relation to task‐related neurotransmitter (GABA/GLx) changes (reactivity) in the mPFC. Therefore, encoding‐related functional connectivity in the mPFC–thalamus–hippocampus network was assessed as a function of subsequent memory and its relation to GABA/GLx reactivity.

## 2 METHODS

### 2.1 Subjects

Twenty‐seven healthy subjects participated (18 females, all right‐handed, mean age 23.1 years). None of the subjects used any medication, had a history of neurological or psychiatric illness, drug abuse, or head trauma. The study was approved by the local medical ethics committee and written informed consent was obtained for each subject.

### 2.2 Experimental design

**Figure 1:**
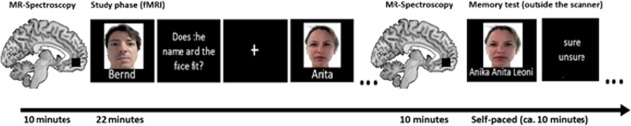
Illustration of the experimental design. Before and after the study phase (fMRI), GABA and GLx in the mPFC (black square) were assessed with single‐voxel MR spectroscopy (MRS). After scanning, subjects performed a cued‐recall memory test [Color figure can be viewed at http://wileyonlinelibrary.com]

Subjects underwent initially an MRS scan of a single voxel positioned in the mPFC, which was followed by the study phase of a face name association task during which whole‐brain functional images were acquired. Thereafter, a second MRS session of the same mPFC voxel was carried out. After scanning, subjects performed a memory test (Figure 1).

### 2.3 Face–name association task

During fMRI, 120 photographs of unknown faces (half males) uniquely associated with names written underneath were sequentially displayed at the center of the screen for 2 s each (mean ISI: 6.7 s; range: 3–10 s). The faces were standardized according to several criteria such as no strong emotional facial expression, direct gaze contact, no glasses, no beard, no headdress, and so on, and the length of names ranged from 3 to 8 letters (mean length ± *SD* = 5.04 ± 1.23). Subjects were explicitly instructed to memorize the face–name associations for a subsequent memory test. To encourage elaboration of the stimuli, subjects were asked to judge whether the face fitted well with the name or not. The face–name trials were intermixed with 120 trials (each 2 s) of visual fixation (null event). The fixation (and null event) stimulus was a white fixation cross‐centered on a black background. After the second MRS session, subjects completed a cued‐recall memory test for all 120 face–name associations outside the scanner. Here, each face was presented on the screen, now shown with three names printed in white letters below the face; the correct name that was actually paired with the face during encoding, and two incorrect names which were originally paired with different faces during encoding. Subjects were instructed to indicate which name was correctly associated during study and whether they had high or low confidence that they made the correct choice.

As behavioral research has shown that the subjective feeling of confidence is related to the recollection of specific episodic details and memory contend vividness (Chua et al., 2004; Robinson, Johnson, & Herndon, 1997; Robinson, Johnson, & Robertson, 2000; Robinson & Johnson, 1996), we assume that the level of confidence reflects the strength and/or associative richness of the retrieved memory. In this regard, we assume that associations remembered with high confidence reflect strong memories, whereas associations remembered with low confidence reflect a mixture of familiarity and guesses that occur by chance (Chua et al., 2004; Sperling et al., 2003; Otten, Henson, & Rugg, 2001), which would be functionally equivalent to incorrect responses (Section 3.1). Therefore, memory performance was determined by the number of high confident remembered minus the number incorrect plus the number of low confident remembered associations.

### 2.4 MR data acquisition

Scanning was performed using a 3 T (Magnetom Trio TIM 3 T, Siemens, Munich) scanner with a 32‐channel head‐coil. Before MRS acquisition, a three‐dimensional T1‐weighted structural image was acquired to guide voxel placement (MPRAGE; FOV  =  240 mm × 240 mm, TR = 2300 ms, TI = 1100 ms, TE = 3.03 ms, 192 slices, spatial resolution = 1 × 1 × 1 mm3, flip angle = 8 degrees). Afterwards, single voxel edited 1H‐MR Spectra from the left ventral medial prefrontal cortex (20 × 20 × 20mm) were acquired before and after the fMRI task. We performed a unilateral analysis to avoid partial volume effects caused by midline CSF and we chose the left hemisphere since face–name association learning involves in particular left prefrontal cortices (Chua et al., 2004). To ensure that MRS voxel positioning before and after fMRI were not distorted by subject movements, an auto alignment sequence (AAScout) were run before the structural scan and before each MRS acquisition. The AAScout takes two low‐resolution whole‐brain scans which were utilized to positioning the subject brain according a vendor brain atlas on the scanner. Moreover, after positioning of the first MRS voxel according to anatomical landmarks (left mPFC, below the bent of corpus callosum), the positioning parameters were stored and used for the second MRS voxel positioning. To ensure goodness of fit, any cases where shimming resulted in FWHM >20 Hz were rejected. Single‐volume, MEGA point‐resolved spectroscopy (PRESS), J‐difference editing sequence was used to measure GABA and glutamate/glutamine (Mescher, Merkle, Kirsch, Garwood, & Gruetter, 1998). For each spectrum (edit on and edit off), 128 spectral scans were acquired (TR = 1600 ms, TE = 68 ms,). For functional MRI, we acquired with ascending slice acquisition a T2*‐weighted echo‐planar imaging sequence (31 axial slices; volume repetition time (TR), 2.39 s; echo time (TE), 9.4 ms; 90° flip angle; slice matrix, 64 × 64; slice thickness, 3.0 mm; field of view, 1344 × 1344mm).

### 2.5 MRS data processing

**Figure 2:**
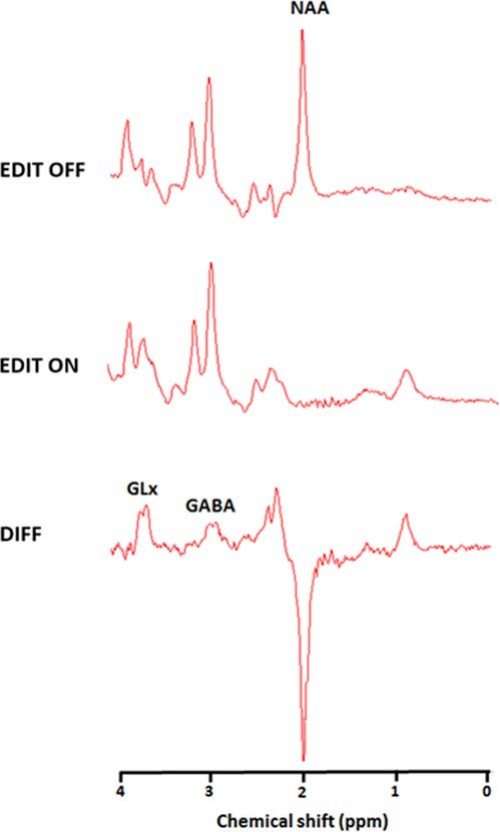
The figure shows the proton magnetic resonance spectroscopy spectra with the editing radiofrequency pulse off (top panel) and on (middle panel). With the editing pulse off, a standard spectroscopy spectrum is obtained, allowing quantification of the *N*‐acetylaspartate (NAA) peak. The spectrum on the bottom is the difference of the spectra “edit off” minus “edit on” yielding the γ‐aminobutyric acid (GABA) and glutamate‐glutamine (Glx) peaks [Color figure can be viewed at http://wileyonlinelibrary.com]

The difference spectra were calculated by subtracting the edit on and edit off spectra. The ratio of GABA and glutamate/glutamine (GLx) to NAA was determined using the AMARES package (Vanhamme, Sundin, Hecke, & Huffel, 2001) that is integrated within the jMRUI software (Naressi, Couturier, Castang, de Beer, & Graveron‐Demilly, 2001). We have chosen to calculate the ratio to relative NAA and not creatine, because GABA and creatine resonate at similar frequencies. As a consequence, the creatine peak in the “edit off spectrum” is a summation of GABA and creatine and a reduction/increase of GABA would also change the creatine peak in the “edit off spectrum” accordingly. Prior to fitting in jMRUI, all spectra were apodized with a 5 Hz Lorenzian filter (see Figure 2 for a representative edited spectrum). Whereas NAA was modeled from the “edit off spectra” as a single Lorentzian peak, GABA and GLx were modeled from the “different spectra” as a pair of Lorentzian peaks with the same line width as NAA. To probe memory‐related changes in GABA and GLx concentrations, repeated measures ANOVA's were conducted with GABA/NAA and GLx/NAA levels before versus after the fMRI task as inputs. Moreover, we used the difference scores (MRS2 − MRS1) to evaluate the relation of encoding‐related neurotransmitter reactivity with memory performance and functional connectivity.

### 2.6 MRI data preprocessing

Using SPM8 (http://www.fil.ion.ucl.ac.uk/spm/software/spm8), functional images were realigned, and the subject mean was coregistered with the corresponding structural MRI by using mutual information optimization. These images were subsequently slice‐time corrected, spatially normalized and transformed into a common space, as defined by the Montreal Neurological Institute (MNI) T1 template, as well as spatially filtered by convolving the functional images with an isotropic 3D Gaussian kernel (8 mm^3^ full‐width at half‐maximum).

### 2.7 fMRI analysis (determination seed voxels for PPI)

In line with our primary aim, we probed mPFC–thalamus–hippocampus network properties (as measured with PPI) by contrasting successfully remembered with high confidence over low confident remembered/incorrect. Therefore, the explanatory variables (onsets for high confident remembered; low confident remembered/incorrect; judgement and null events [fixation cross]) were temporally convolved with the canonical hemodynamic response function along with its temporal derivatives provided by SPM8. For the statistical analysis, appropriate contrast parameter images were generated for each subject by weighting high confident remembered with 1 and low confident remembered/incorrect with −1, respectively. Subsequently, these contrast images were subjected to a second‐level random effects analysis in which the brain map was initially thresholded with *p* = .001 with a test statistic on cluster‐size thresholded at *p* ≤ .05 (family‐wise error corrected). As we were interested in encoding‐related effects on GABA/GLx concentrations in the mPFC and its relation to functional connectivity in the mPFC–thalamus–hippocampus network, we used the memory‐related brain activation cluster that overlaid with the area of the MRS voxel position as seed region for the subsequent PPI analyses.

### 2.8 Functional connectivity analysis

We performed a PPI analysis from the seed region. The PPI analysis probed encoding related functional connectivity for face–name pairs that were subsequently remembered with high confidence versus low confident remembered/incorrect.

For the PPI analyses, the first eigenvariate of the time course within the seed region was extracted as the physiological factor. Then, the psychological factors were computed. Here, the onset times for high confident remembered and low confident remembered/incorrect were temporally convolved with the canonical hemodynamic response function (weighted with +1 for high confident remembered and −1 for low confident remembered/incorrect). Finally, the interaction factors (PPI) were calculated as an interaction term of the physiological and psychological factors. In the last step, a GLM was conducted with the PPI regressors (physiological, psychological, and interaction factors) of the seed region together with the other experimental condition regressors (onsets for high confident remembered; low confident remembered/incorrect; judgment and null events [fixation cross]), temporally convolved with the hemodynamic response function along with its temporal derivatives, and the six motion regressors derived from realignment parameters during preprocessing of the functional scans.

To probe whether functional connectivity is related to neurotransmitter reactivity in the mPFC, the subject‐specific contrast images for the interaction term (PPI.ppi) were used as inputs for a second‐level regression analyses with the task‐related changes in neurotransmitter level (MRS after‐MRS before) as covariate of interest. We used the difference score as covariate to assess whether neurotransmitter concentration changes, due to memory related processes, predict functional connectivity in the mPFC–thalamus–hippocampus network. For statistical testing, we initially thresholded the whole brain map with *p* = .001 to provide input for our test statistics on cluster‐size threholded at *p* ≤ .05 (family‐wise error corrected). Given our *a priori* interest in the mPFC–thalamus–hippocampus network, we reduced our search space by applying small‐volume correction within these brain regions in all PPI analyses applying the same statistical threshold (*p* ≤ .05, family‐wise error corrected).

### 2.9 Correlation analysis

Pearson's partial correlations analyses were performed by means of SPSS (IBM 21) software to assess the relations between memory performance and neurotransmitter and functional connectivity. As aforementioned, memory performance was determined by the amount of high confident remembered minus the amount of low confident and incorrect remembered associations. Regarding the neurotransmitter, we used the difference scores (MRS2 − MRS1) as input to evaluate the relation of encoding‐related neurotransmitter reactivity and memory performance. For functional connectivity, we extracted the mean beta values of the functional connectivity clusters that reached statistical significance. For this purpose, we used MarsBaR toolbox (Brett, Anton, Valabregue, & Poline, 2002) to create separate masks of the significant clusters which in turn were used in the REX toolbox to extract the mean functional connectivity beta‐values of each cluster for all subjects. We used Bonferroni correction for multiple testing whenever applicable.

## 3 RESULTS

### 3.1 Memory performance

Subjects correctly identified the name associated with the face with high confidence in 36% and with low confidence in 27% of the trials. In 37% of the trials, the subjects were incorrect. One‐sample *t* test revealed that performance for high confidence remembered (high‐confidence hit minus high‐confidence false alarm: *t*(26) = 8.23, *p* < .0001) was above chance level, whereas low confident remembered was at chance level (low confidence hit minus low confidence false alarm: *t*(26) = −0.36, *p* =.724). As low confident responses appeared at chance level, they are likely not associated with the recollection of specific episodic details but rather guessing (Chua et al., 2004; Sperling et al., 2003; Otten et al.,^2001^). Therefore, we used the contrast high confident remembered versus low confident remembered plus incorrect in all analyses. Regarding confidence rating, we could not find a tendency that some participants always have chosen the extreme cases as both the low and the high confident ratings were normally distributed.

### 3.2 MRS analyses

**Figure 3:**
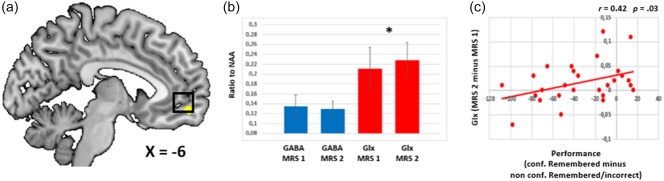
(a) The functional map is overlaid on the MRIcron template brain (ch256). The brain region (yellow cluster) in the mPFC that showed increased memory related brain activation is depicted. The black square indicates the mean position of the MRS voxel, which overlaps with fMRI voxels of the activation cluster. Therefore, this activation cluster served as seed region for the PPI analyses. (b) Concentrations of GABA and GLx before and after the fMRI task are depicted, whereas GABA did not change, Glx concentration increased over time. (c) The Increase in GLx (MRS 2 − MRS 1) correlated positively with memory performance (high confident remembered minus low confident remembered/incorrect) [Color figure can be viewed at http://wileyonlinelibrary.com]

The mean voxel position (center) was *x* = −6, *y* = 44, *z* = −7 in MNI space with a mean percentage of 53% (*SD* = .089) gray matter, 40% (*SD* = .096) white matter, and 7% (*SD* = .025) CSF. First, we assessed whether NAA levels remained stable as assumed. A repeated measures ANOVA comparing NAA levels before and after study revealed no significant change (*F*(1,26) = .17, *p* = .85). Subjecting GLx/NAA ratios of MRS 1 (before study) and MRS 2 (after study) to a repeated measures ANOVA revealed a significant increase (*F*(1,26) = 7.42, *p* = .011), showing that the face–association encoding task was associated with an increase of medial prefrontal GLx concentration. An analog repeated measures ANOVA with GABA/NAA ratios as input did not reveal any significant change (*F*(1,26) = .24, *p* = .62, Figure 3B).

### 3.3 fMRI analyses

**Figure 4:**
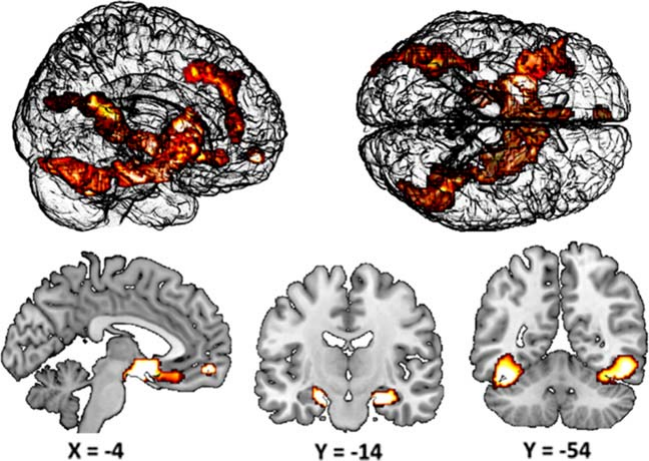
The functional maps are overlaid on the MRIcron template brain (ch256). The images show the significant brain activation. Brain activation was modeled by weighting high confident remembered over low confident remembered/incorrect trials [Color figure can be viewed at http://wileyonlinelibrary.com]

We contrasted high confident remembered over low confident remembered plus incorrect to reveal a seed region for the PPI analyses. Given our *a priori* interest, we reduced our search space to the mPFC–thalamus–hippocampus network by applying small‐volume correction within these brain regions. In this analysis, we found a significant cluster in the mPFC (maxima = MMI −6 54 −10; *p* = .03; Figure 4) that overlapped with the location of the MRS voxel (Figure 3A). Note, this activation cluster represents the significant mean group effect and is just a measure of central tendency over all participants (Seghier & Price, 2016). This mPFC cluster was subsequently used as seed region for the PPI analyses. The whole‐brain analysis revealed differential activation in several brain regions (Figure 4) as, for instance, the hippocampus (maxima = MMI 30 −12 −18; *p* < .001; maxima = MMI −20 −22 −18; *p* < .001 Figure 4), fusiform gyrus (maxima = MMI 42 −46 −14; *p* < .001; maxima = MMI −40 −54 −20; *p* < .001; Figure 4) and left prefrontal cortex (maxima = MMI −34 34 −6; *p* < .001; maxima = MMI −40 −24 20; *p* < .001; Figure 4)

### 3.4 Psychophysiological interaction

**Figure 5:**
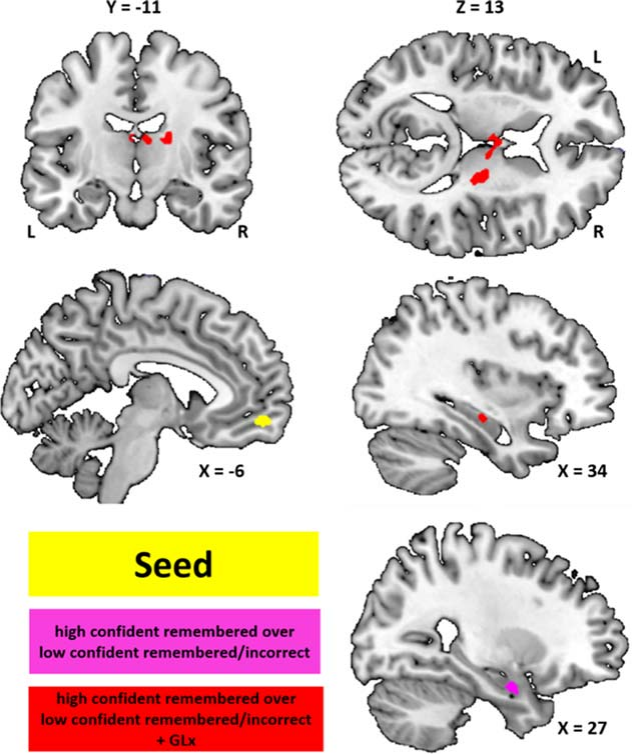
The functional maps are overlaid on the MRIcron template brain (ch256). The images show the different functional connectivity's in the mPFC–thalamus–hippocampus network. The yellow cluster in the mPFC indicates the seed region for the PPI analyses. The purple cluster indicate functional connectivity between the mPFC and the right hippocampus (high confident remembered vs low confident remembered/incorrect) not related to GLx. The red clusters indicate functional connectivity between the mPFC and thalamus (high confident remembered vs low confident remembered/incorrect +GLx) and right hippocampus that is related to the change in GLx concentration (the more GLx increase the more functional connectivity). +GLx = covariate GLx (different score) [Color figure can be viewed at http://wileyonlinelibrary.com]

Contrasting encoding‐related mPFC connectivity pattern for face–name pairs subsequently remembered with high confidence compared to low confidence/incorrect revealed stronger connectivity to right hippocampus (maxima = MMI 28 −4 −22; *p* = .05). When taking the GLx/NAA difference scores as covariates of interest (high confident remembered compared to low confident remembered/incorrect + GLx), we found a positive relation between the GLx/NAA different score and mPFC–thalamic connectivity (ventral anterior nuclei: maxima = MMI −6 10 14/8 −14 14; *p* = .025; dorsal margin: maxima = MMI 20 −16 16; *p* = .016; Figure 5). Moreover, we found a positive relation between the GLx/NAA different score and mPFC–hippocampus connectivity (maxima = MMI 34 −18 −12; *p* = .05). Thus, the more GLx/NAA increased over the memory task, the stronger the mPFC−thalamic and mPFC−hippocampus functional connectivity.

In a next step, we assessed whether the GLx reactivity and the functional connectivity parameters are relevant for memory performance. Using Pearson's partial correlation, we found a positive correlation between the GLx/NAA difference scores and memory performance across subjects (*r* = .42; *p* = .03; Figure 3C). Hence, those individuals with the highest increase in mPFC GLx concentration were those that preformed best and vice versa. Moreover, we found a positive correlation between memory performance and the mPFC connectivity parameters (mean *b* values) of one thalamic cluster (dorsal margin: *r* = .39; *p* = .04) and the hippocampus cluster (*r* = .56; *p* = .004) that revealed a positive relation between the GLx different score and functional connectivity (high confident remembered over low confident remembered/incorrect + GLx; Table 1).

**Table 1:** The correlations between memory performance and the different functional connectivity's (Figure 4) are depicted

Functional connectivity		Performance
High conf. remembered over low conf. remembered/incorrect	(mPFC − right hippocampus)	*r* = .24
High conf. remembered over low conf. remembered/incorrect + Glx	(mPFC − right hippocampus)	*r* = .56^b^
High conf. remembered over low conf. remembered/incorrect + Glx	(mPFC − dorsal margin of thalamus)	*r* = .39^a^
High conf. remembered over low conf. remembered/incorrect + Glx	(mPFC − ventral anterior nucleus of thalamus)	*r* = .22

The GLx/NAA reactivity (MRS 2 − MRS 1)‐related functional connectivities of mPFC to the dorsal margin of thalamus (high confident remembered over low confident remembered/incorrect +GLx) and right hippocampus correlate positive with memory performance.

a Uncorrected significant.

b Significant after Bonferroni correction.

**Figure 6:**
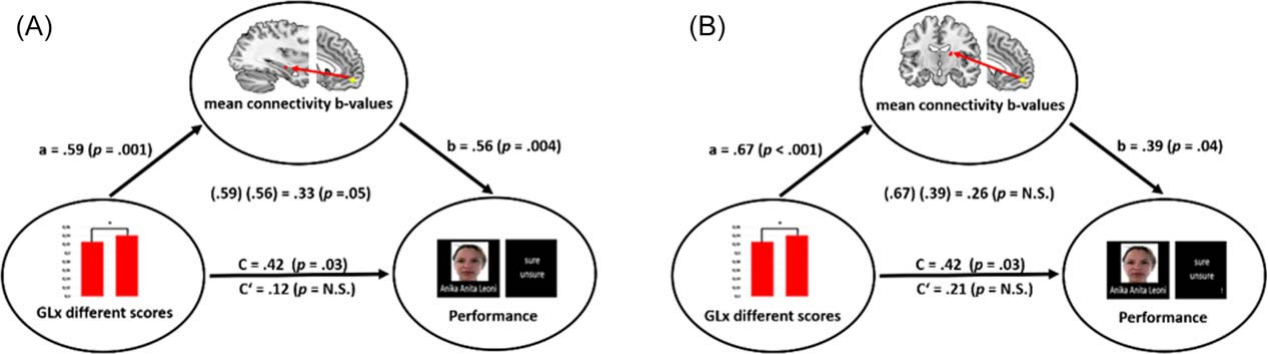
The outcome of the mediation analyses. (a) The diagram shows that the relationship between GLx reactivity (GLx/NAA different scores) and memory performance was mediated by functional connectivity between mPFC and hippocampus (high confident remembered vs low confident remembered/incorrect + GLx). GLx different scores were positively associated with mPFC/hippocampus functional connectivity (apath). MPFC/hippocampus functional connectivity predicted memory performance (b‐path). GLx different scores directly predicted memory performance (c‐path) but if controlled for the mediator this effect diminished (c'‐ path). The indirect effect (a, b) was .33 which was statistically significant (Sobel test). (b) The diagram shows that the relationship between GLx reactivity (GLx/NAA different scores) and memory performance seems also mediated by functional connectivity between mPFC and thalamus (high confident remembered vs low confident remembered/incorrect + GLx); however, the indirect effect (a)(b) =.26 was not statistically significant [Color figure can be viewed at http://wileyonlinelibrary.com]

To integrate these findings further, we assessed the relationship between the significant correlations by utilizing mediation analysis (Hayes, 2013). We performed two separate analyses to test whether the GLx related mPFC–thalamus (dorsal margin) and/or mPFC–hippocampus functional connectivity mediated the association between GLx reactivity and memory performance. Regarding mPFC–thalamus (dorsal margin) functional connectivity (Figure 6B), we found that the standardized regression coefficients between GLx reactivity and memory performance (c‐path), GLx reactivity, and functional connectivity (a‐path) and functional connectivity and memory performance (b‐path) were statistically significant (Figure 6B). The standardized indirect effect (*a* × *b*) was (.67)(.39) = .26. We tested the significance of this indirect effect using bootstrapping procedures via the PROCESS macro for SPSS (PROCESS v2.16, Hayes 2016). Standardized indirect effects were computed for each of 10,000 bootstrapped samples, and the 95% confidence interval was computed by determining the indirect effects at the 2.5th and 97.5th percentiles. The bootstrapped standardized indirect effect was .26, and the 95% confidence interval ranged from −.02 to .69. The indirect effect was statistically not significant (*p* = .29) as tested with the Sobel test, implemented in the PROCESS macro. Regarding mPFC–hippocampus functional connectivity (Figure 6A), we found also a significant a‐path and b‐path. The standardized indirect effect (*a* × *b*) was (.59)(.56) = .33 (95% confidence interval ranged from .03 to .68) which was statistically significant (*p* = .05). Thus, the positive association between GLx reactivity (GLx/NAA different scores) and memory performance seems to be mediated by functional connectivity between mPFC and hippocampus.

## 4 DISCUSSION

This study aimed at elucidating the role of the human mPFC–thalamus–hippocampus network in associative memory formation and its relation to neurotransmitter reactivity. Therefore, encoding‐related mPFC concentration changes of GABA and GLx were assessed and related to memory performance and memory‐related functional connectivity in the mPFC–thalamus–hippocampus network. In the following, we discuss first the GABA/Glx reactivity in the mPFC, its relation to memory performance and then its relation to the mPFC–thalamus–hippocampus network.

Whereas metabolite concentrations at rest have been frequently assessed, the dynamics of GABA and GLx during task performance have been less frequently investigated (Kühn et al., 2016). Here we show that GLx but not GABA increased in the mPFC when subjects memorized face–name associations. This finding complements that of Hertz et al. (2003) who showed increased forebrain concentrations of glutamate and glutamine after passive avoidance learning in animals. Notably, the authors found also a reduction in glycogen concentrations, which suggest increased de novo synthesis of glutamate/glutamine from glucose or glycogen during learning (Hertz et al., 2003). However, what may be the function of increasing glutamate and glutamine? Glutamine is the metabolic precursor of glutamate in the mammalian brain (Petroff, 2002). After synaptic transmission of glutamate, surrounding glia cells metabolize glutamate to glutamine, which is in turn transported to the neurons were it is synthesized to glutamate (Petroff, 2002). Thus, one may speculate that the increase in glutamate/glutamine is related to an increased need of synaptic glutamate to maintain sustained glutamatergic transmission. It is generally accepted that glutamatergic transmission results in LTP a leading candidate for the neurophysiological substrate of learning and memory (Myhrer, 2002). Manipulating glutamatergic receptors appear to affect LTP and memory. For instance, blocking glutamatergic transmission via an NMDA receptor antagonist causes LTP reduction and impaired learning (Davis et al.,^1992^; Stäubli et al.,^1994^). Therefore, the assumption of increased glutamatergic transmission would be in line with our finding regarding memory performance. We found that individuals with a higher increase in GLx remembered more associations with high confidence. Thus, it is possible that the GLx increase reflects enhanced glutamatergic transmission that resulted in increased synaptic plasticity and therefore better memory performance. To summarize, memorizing face–name associations increased GLx concentrations in the mPFC and this increase was associated with better subsequent memory suggesting that the mPFC GLx reactivity is functionally important for memory formation and not merely a reflection of neural processing.

Previous studies reported a decrease in cortical GABA concentrations when subjects learned novel motor skills (Floyer‐Lea et al., 2006; Sampaio‐Baptista et al., 2015) suggesting a potential role of GABA reactivity in associative learning. In this study, however, we did not observe such a reduction in mPFC GABA concentrations. The finding that GABA levels remained stable may be related to the timeframe between the two MRS measurements. For instance, Michels et al. (2012) showed an initial increase in frontal GABA that gradually decreased during a working memory task suggesting time as an important variable. Moreover, Floyer‐Lea et al. (2006) reported a 5% reduction of GABA after 30 min but 18% after 50 min of motor learning, whereas Kühn et al. (2016) found increased GABA in the ACC after the engagement in an interference task for 13 min. Thus, it is possible that our second MRS measurement (22 min after baseline) was at a time point when GABA has gradually “recovered” from an initial increase. Therefore, future studies should measure the mPFC GABA/GLx dynamics on multiple time points during associative learning.

Regarding network properties, we discuss first the functional connectivity and then the functional connectivity associated to GLx reactivity. If seeded from the mPFC, we found increased functional connectivity to the right anterior hippocampus for associations later remembered with high confidence compared to low confident remembered/incorrect. This functional connectivity, however, did not predict memory performance. With other words, subjects that had a stronger functional mPFC–hippocampus (anterior) connectivity did not remember more face–name associations.

Concerning mPFC GLx reactivity, we found a positive association between mPFC GLx increase and mPFC functional connectivity to both the thalamus and the hippocampus. This findings are in line with rodent data showing that ketamine (NMDA receptor antagonist) alters functional connectivity between several mPFC regions and thalamic nuclei and hippocampus (Dawson et al., 2015; Dawson, Morris, & Pratt, 2013).

With respect to the mPfC–thalamic functional connectivity, we found increased GLx (reactivity)‐related functional connectivity to the ventral anterior nuclei (bilateral) and to the dorsal margin of the thalamus. Regarding the ventral anterior nuclei, our findings are in line with the model proposed by Aggleton et al. (2011) and Aggleton & Brown (1999). These authors proposed that anterior thalamic nuclei, as the ventral anterior nucleus, and its connections to the hippocampus are essential for recollection based recognition. In addition, the authors indicated that prefrontal cortices interact with this thalamic–hippocampal system, engaging in efficient encoding strategies that may aid subsequent recollection (Aggleton & Brown, 1999). With respect to the functional connectivity to the dorsal margin of the thalamus, albeit speculative, one may assume that this thalamic region represents the thalamic reticular nucleus (Viviano and Schneider, 2015; Zikopoulos and Barbas, 2006; Jones, 1985), a layer of GABAergic cells wrapping the dorsolateral segments of the thalamus (Jones, 1985; Zikopoulos and Barbas, 2006). GABAergic neurons in the thalamic reticular nucleus modulate both corticothalamic and thalamocortical communications (Slotnick, Moo, Kraut, Lesser, & Hart, 2002; Lozsádi, 1995; Steriade, Contreras, Curró Dossi, & Nuñez, 1993; Jones, 1985). For instance, the thalamic reticular nucleus receives excitatory inputs from cortex and other thalamic nuclei and sends inhibitory projections (GABAergic) back to thalamic nuclei thereby controlling the activity/oscillations in thalamocortical loops (Slotnick et al., 2002; Lozsádi, 1995; Steriade et al.,^1993^). Brain oscillations result in synaptic plasticity via synchronizing and desynchronizing neural assemblies and are therefore one of the core mechanisms underlying episodic memory (Hanslmayr, Staresina, & Bowman, 2016). These, specific functions of the thalamic reticular nucleus may explain the finding that GLx related mPFC–thalamic reticular nucleus functional connectivity predicted memory performance. In detail, we found that individuals with higher mPFC–thalamic reticular nucleus functional connectivity beta‐values remembered more associations with high confidence jwcompared to low confident remembered/incorrect. With respect to GLx (reactivity) related mPFC–hippocampus functional connectivity, we found increased functional connectivity to the right hippocampus. This GLx (reactivity)‐related mPFC–hippocampus functional connectivity predicted not only memory performance but also appeared to mediate the positive association between GLx reactivity and memory performance. Together, we found that the task‐related change in mPFC GLx concentration was associated with mPFC functional connectivity to both the thalamus and hippocampus, which appeared to be functionally relevant in terms of memory performance.

In summary, this study demonstrates that mPFC GLx levels increased during associative memory encoding, which was positively associated with memory performance. Moreover, we found that mPFC GLx reactivity appeared to modulate functional connectivity within the mPFC–thalamus–hippocampus network. Subjects with a higher increase in GLx were those that had stronger functional connectivity in this network for associations subsequently remembered with high confidence compared to low confident remembered/incorrect. In addition, this GLx‐related functional connectivity was associated with better memory for the face–name associations and appeared to mediate the positive association between GLx reactivity and memory performance. These findings suggest that mPFC–thalamic–hippocampus interactions during encoding are related to neurotransmitter reactivity, which might facilitate the formation of detailed and vivid memories.

## CONFLICT OF INTEREST

The authors declare no conflict of interest
